# Microchemical Techniques for Multiclass Fungicide Residue Analysis in Complex Food Matrices

**DOI:** 10.3390/foods15142467

**Published:** 2026-07-12

**Authors:** Steven Suryoprabowo, Andreas Romulo, Eddy Seong Guan Cheah, Yahui Guo

**Affiliations:** 1 Food Technology Department, Faculty of Engineering, Bina Nusantara University, Jakarta 11480, Indonesia; andreas.romulo@binus.edu; 2 Department of Biological Science, Faculty of Science, Universiti Tunku Abdul Rahman, Jalan Universiti, Bandar Barat, Kampar 31900, Perak, Malaysia; cheahsg@utar.edu.my; 3 School of Food Science and Technology, Jiangnan University, No. 1800 Lihu Avenue, Wuxi 214122, China; guoyahui@jiangnan.edu.cn

**Keywords:** fungicide residues, food safety monitoring, microchemical extraction, pesticide residue analysis, sustainable analytical chemistry

## Abstract

Fungicide residues in complex food matrices represent an increasingly important challenge in food safety monitoring because intensive agricultural practices, diverse fungicide chemistries, and tropical production conditions can generate multiclass contamination patterns, particularly in Southeast Asian food systems. This review critically evaluates literature published between 2019 and 2026 on microchemical analytical strategies for multiclass fungicide residue determination in fruits, vegetables, rice, spices, and processed foods. The review focuses on the integration of miniaturized and green sample preparation techniques, including modified QuEChERS, dispersive liquid–liquid microextraction, solid-phase microextraction, hollow-fiber liquid-phase microextraction, magnetic solid-phase extraction, and deep eutectic solvent-based extraction, with advanced chromatographic and mass spectrometric platforms. Current evidence shows that these methods can reduce solvent consumption, improve analytical efficiency, and support sensitive residue determination when coupled with UHPLC–MS/MS, GC–MS/MS, and high-resolution mass spectrometry. However, method performance remains strongly matrix-dependent and is constrained by matrix effects, limited standardization of emerging extraction materials, inconsistent validation practices, and trade-offs among selectivity, throughput, cost, and sustainability. No single extraction strategy is universally optimal for all food matrices or fungicide classes. Future research should therefore prioritize matrix-adapted hybrid workflows, harmonized validation protocols, improved detection of transformation products, and broader use of high-resolution screening strategies to support reliable, sustainable, and regulatory-compliant fungicide residue monitoring.

## 1. Introduction

Fungicides constitute a major class of agrochemicals whose widespread use presents significant analytical challenges due to their structural diversity, wide polarity range, and frequent co-occurrence in complex food matrices. The increasing global use of fungicides has led to complex residue patterns in food systems, requiring analytical methods capable of simultaneous multiclass detection at trace levels. Major fungicide classes (e.g., azoles, strobilurins, and SDHIs) exhibit diverse physicochemical properties that directly influence extraction efficiency and detection performance in multiresidue analytical workflows. Their deployment spans pre-harvest foliar spraying, seed treatment, and post-harvest preservation to reduce spoilage and mycotoxin development [1]. Recent global surveillance programs confirm that fungicides account for a significant proportion of pesticide residues detected in food monitoring studies. Multiresidue investigations across Europe, Asia, and Africa consistently report high detection frequencies of azoxystrobin, tebuconazole, boscalid, carbendazim, and difenoconazole in fresh produce [2]. Climate variability, especially increased humidity and temperature fluctuations, has further amplified fungal disease prevalence, prompting intensified fungicide application strategies. Consequently, residue surveillance systems face growing analytical demands for simultaneous detection of multiple fungicide classes at trace levels. From an analytical perspective, the occurrence of fungicide residues at trace levels necessitates highly sensitive and selective detection strategies to ensure compliance with regulatory limits. Although toxicological concerns exist, the primary analytical challenge lies in detecting these residues at trace levels across complex matrices with high selectivity and accuracy. Regulatory bodies such as the Codex Alimentarius Commission and European Food Safety Authority (EFSA) establish maximum residue limits (MRLs) to safeguard public health, necessitating reliable analytical monitoring tools capable of achieving sub µg/kg detection limits.

Southeast Asia (SEA) provides analytically challenging matrices due to high moisture content, complex plant metabolites, and intensive multiclass pesticide usage, which collectively complicate residue determination. The region’s tropical climate characterized by high humidity, monsoon cycles, and elevated temperatures creates ideal conditions for fungal proliferation in crops such as rice, oil palm, banana, mango, durian, chili, and leafy vegetables. To mitigate yield losses, farmers frequently adopt intensive fungicide spraying regimes throughout growing seasons. Residue monitoring studies conducted in Thailand, Vietnam, Indonesia, Malaysia, and the Philippines indicate increasing detection frequencies of multiclass fungicide residues in vegetables and fruits destined for both domestic consumption and export markets. Several studies report multiple residues per sample, often exceeding three or more fungicides in a single commodity [3]. In tropical horticultural systems, rotational use of different fungicide classes to prevent resistance contributes to this multiclass contamination pattern. Export-oriented agriculture further complicates the scenario. Southeast Asian producers must comply with stringent MRLs imposed by importing regions such as the European Union and Japan. Variability in regulatory standards among trading partners increases the need for high-sensitivity, harmonized analytical methods capable of detecting fungicides across a broad polarity and stability spectrum.

**Figure 1:**
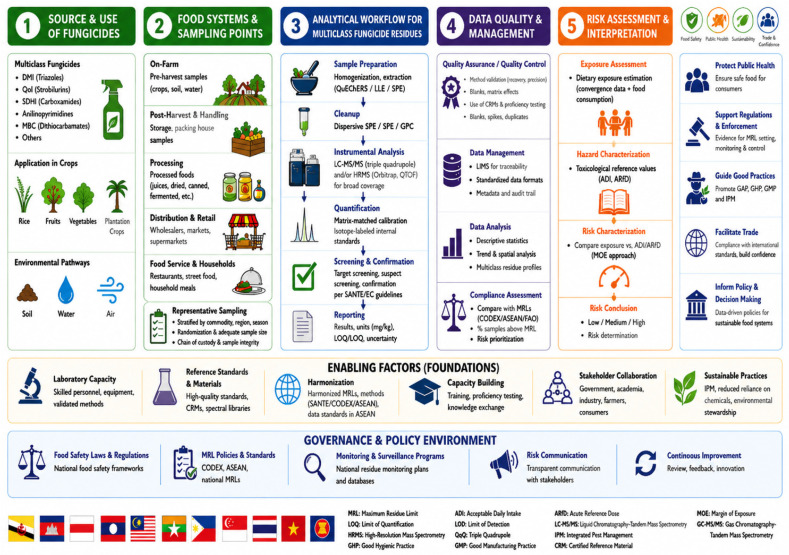
Conceptual framework of multiclass fungicide residue analysis in Southeast Asian food systems.

Recent residue-monitoring literature indicates a clear transition from single-analyte detection toward comprehensive multiclass pesticide and fungicide surveillance, because food samples frequently contain combinations of azoles, SDHIs, strobilurins, benzimidazoles, and relevant transformation products [2]. This shift reflects integrated disease-management practices, rotational fungicide use, and increasingly complex residue profiles in tropical agricultural commodities. Conventional approaches based on exhaustive liquid–liquid extraction or solid-phase extraction, followed by GC or LC detection, have provided the foundation for regulatory monitoring [4]; however, these methods often require high solvent volumes, extended sample preparation, and matrix-specific optimization [5]. These limitations become more critical when laboratories must detect chemically heterogeneous fungicide classes at trace levels while controlling matrix effects, improving throughput, and reducing environmental burden. Although recent advances in microchemical extraction and chromatographic–mass spectrometric detection have improved sensitivity and sustainability, existing reviews rarely integrate sample preparation, instrumental compatibility, matrix complexity, and validation requirements into a unified analytical framework. Therefore, this review critically evaluates microchemical extraction strategies for multiclass fungicide analysis, assesses their compatibility with advanced chromatographic and mass spectrometric platforms, identifies key challenges in tropical food matrices, and proposes future directions for sustainable, high-throughput, and regulatory-compliant residue monitoring [1]. The overall analytical workflow linking fungicide application, residue occurrence, sample preparation, and instrumental detection is summarized in Figure 1. Despite these advances, a critical methodological gap remains: most existing analytical strategies optimize either extraction efficiency or detection sensitivity, but rarely both across chemically heterogeneous fungicide classes under a unified framework. This review therefore uniquely focuses on the mechanistic source of analytical difficulty physicochemical incompatibility across fungicide classes and systematically evaluates how microchemical techniques attempt to resolve this constraint across complex food matrices.

## 2. Methodology

This review was conducted as a structured literature review following a transparent identification, screening, eligibility, and synthesis procedure to evaluate microchemical strategies for multiclass fungicide residue analysis in complex food matrices. The literature search covered publications from January 2019 to December 2026 and was conducted in Scopus, Web of Science Core Collection, ScienceDirect, PubMed, and Google Scholar. The final search was performed using the following Boolean strings, adjusted only for database-specific syntax: (“fungicide residue*” OR “multiclass fungicide*” OR “multiresidue fungicide*” OR “pesticide residue*”) AND (“food matrix” OR “complex food matrix” OR fruit* OR vegetable* OR rice OR spice* OR “processed food*”) AND (“microchemical extraction” OR microextraction OR QuEChERS OR “mini-QuEChERS” OR DLLME OR “dispersive liquid–liquid microextraction” OR SPME OR “solid-phase microextraction” OR MSPE OR “magnetic solid-phase extraction” OR HF-LPME OR “hollow-fiber liquid-phase microextraction” OR “deep eutectic solvent*” OR DES) AND (“LC–MS/MS” OR “UHPLC–MS/MS” OR “GC–MS/MS” OR HRMS OR Orbitrap OR QTOF) AND (“matrix effect*” OR validation OR recovery OR LOD OR LOQ OR “green analytical chemistry” OR sustainability); an additional regional search used (“Southeast Asia” OR Indonesia OR Malaysia OR Thailand OR Vietnam OR Philippines) AND (“fungicide residue*” OR “pesticide residue*”) AND (“food monitoring” OR “food safety”). Google Scholar screening was limited to the first 200 relevance-ranked records to reduce low-specificity retrieval. Records were included when they: reported peer-reviewed analytical studies, regulatory reports, or analytical guidelines published in English; addressed fungicide residues or fungicide-containing multiresidue panels in food matrices; described microchemical or miniaturized sample preparation, including QuEChERS variants, DLLME, SPME, HF-LPME, MSPE, SBSE, PT-SPE, DES-based extraction, or related green extraction approaches; used chromatographic–mass spectrometric detection such as UHPLC–MS/MS, LC–MS/MS, GC–MS/MS, Orbitrap-HRMS, or QTOF; and provided validation or performance data, including recovery, precision, LOD, LOQ, matrix effects, or regulatory applicability. Studies were excluded when they focused only on environmental, biological, or non-food matrices; addressed single-analyte analysis without methodological relevance to multiclass monitoring; lacked validation data; used only conventional extraction without microchemical relevance; were conference abstracts, editorials, patents, theses, or non-English records; or had unavailable full texts. From the final included studies, a subset analysis was conducted to identify SEA-relevant publications. Studies were classified as SEA-relevant if they reported sampling, monitoring, or analytical validation in Indonesia, Malaysia, Thailand, Vietnam, or the Philippines. The proportion of SEA-specific studies was quantified relative to the total eligible dataset to ensure transparency in regional representativeness.

## 3. Major Chemical Classes Detected in SEA Foods

**Figure 2:**
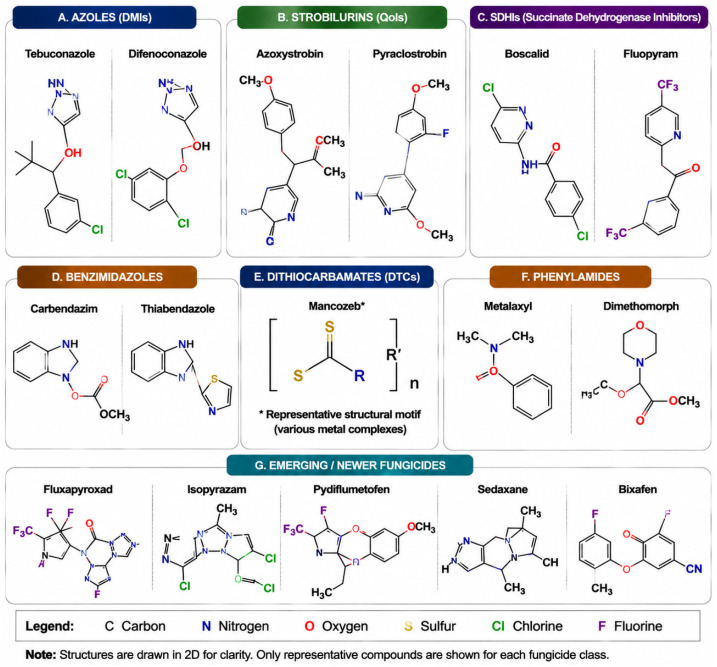
Structural representation of major fungicide classes and representative compounds relevant to multiclass residue analysis in SEA food matrices.

Fungicide classification is analytically significant because differences in chemical structure and physicochemical properties directly influence extraction efficiency, chromatographic separation, and ionization behavior in mass spectrometric detection. Analytical strategies must account for structural diversity, polarity differences, and degradation pathways, which influence extraction behavior, chromatographic retention, and ionization efficiency. Recent monitoring programs across Thailand, Vietnam, Indonesia, Malaysia, and the Philippines consistently report azoles, strobilurins, SDHIs, benzimidazoles, and dithiocarbamates as dominant fungicide residues in fruits, vegetables, rice, and plantation crops. From an analytical standpoint, the coexistence of multiple fungicide classes within a single sample represents a major limitation for conventional multiresidue methods, necessitating the development of more selective and adaptable microchemical strategies. Figure 2 presents the chemical structures of major fungicide classes and representative compounds relevant to multiclass residue analysis in SEA food matrices.

Multiresidue monitoring in SEA requires analytical workflows capable of handling wide polarity ranges, diverse stability profiles, and complex transformation pathways, which challenge conventional extraction and detection methods. These workflows must accommodate wide polarity ranges and stability differences, which often compromise simultaneous extraction and detection in multiresidue methods. In regional market surveys, fungicides are often among the dominant residue categories in plant foods, reflecting intensive disease pressure in humid tropical agroecosystems and frequent prophylactic spray schedules [6]. Method performance is commonly benchmarked against EU SANTE guidance (recoveries typically 70–120% with RSD ≤20% under validated conditions), which is widely adopted even outside Europe as a practical quality-control framework for enforcement-grade data [7].

Azoles especially triazoles (e.g., tebuconazole, propiconazole, difenoconazole, myclobutanil, penconazole) are demethylation inhibitors (DMIs) that block sterol biosynthesis (C14-demethylation), disrupting fungal membrane integrity and growth. Their agronomic popularity in tropical systems stems from broad-spectrum efficacy on leaf spots, anthracnose, mildews, and postharvest diseases, which makes them frequent targets in SEA residue monitoring. In Central Vietnam, difenoconazole was among the higher-frequency detections in vegetable samples, illustrating how DMI use translates into measurable residues at harvest [8]. From an analytical standpoint, many azoles are moderately lipophilic, amenable to LC–ESI–MS/MS (often positive mode), but matrix effects can be substantial in high-pigment or high-essential-oil matrices (e.g., herbs, leafy greens). Method design should therefore include matrix-matched calibration or isotopically labeled internal standards when feasible, and careful evaluation of ion suppression/enhancement [9]. A growing challenge is that “azole fungicides” also implies the need to track relevant metabolites and transformation products; recent reviews highlight expanding interest in rapid/alternative detection strategies and the analytical gaps for azole-containing residues in foods [10].

Strobilurins (QoIs) such as azoxystrobin, pyraclostrobin, trifloxystrobin, kresoxim-methyl, and fluoxastrobin inhibit mitochondrial respiration at the cytochrome bc1 complex (Qo site), causing energy deprivation in fungi. They are widely used on fruits and vegetables, and residue surveys often report high detection frequencies in produce due to repeated applications and relatively persistent parent compounds. In a multiresidue survey of litchi (May 2019–Aug 2020), pyraclostrobin was among the most frequently detected residues, consistent with QoI-heavy disease management in fruit production systems [11]. Analytically, QoIs are typically hydrophobic and well suited to LC–MS/MS (often positive mode), though some can also be measured by GC–MS/MS depending on volatility and method scope. QoIs may undergo environmental and microbial transformation, screening approaches increasingly consider degradation products alongside parents; mechanistic and fate-focused reviews emphasize the ubiquity of strobilurins in agricultural matrices and the importance of understanding transformation pathways that can affect residue profiles [12]. Human biomonitoring evidence is also emerging in the recent literature, reinforcing the need for robust residue methods that can support exposure assessment beyond food matrices alone [13].

SDHIs (e.g., boscalid, fluopyram, fluxapyroxad, penthiopyrad) inhibit succinate dehydrogenase (mitochondrial complex II), impairing fungal respiration. Their use has expanded globally across fruits, vegetables, and plantation crops, and SDHIs are now routine inclusions in multiresidue panels. Recent method papers report highly sensitive LC–MS/MS quantification of multiple SDHIs across diverse foods and beverages, illustrating increasing analytical maturity for this class [14]. For SEA-relevant contexts, SDHI residues are often expected in high-disease-pressure crops (e.g., tomatoes, cucurbits, peppers, grapes, tropical fruits). Fluopyram, for example, has been studied extensively for residue behavior and dietary risk assessment across fruit and vegetable matrices using QuEChERS with chromatographic MS detection [15]. Beyond enforcement, SDHI risk discussions have also prompted national-level scientific assessments (e.g., ANSES) focused on exposure and potential hazards, which further elevates the importance of reliable monitoring data [16]. Analytically, SDHIs are generally LC–MS/MS-friendly but can exhibit matrix-dependent recovery and ionization variability; comprehensive validation across representative SEA matrices (leafy greens, herbs, tropical fruits) is critical [9].

Benzimidazoles including carbendazim, benomyl, thiabendazole, thiophanate-methyl act primarily by disrupting microtubule assembly (β-tubulin binding), inhibiting cell division. Although resistance issues have constrained use in some settings, benzimidazoles remain relevant because of legacy application, postharvest use (e.g., thiabendazole), and the persistence of carbendazim as both parent and metabolite marker. Recent reviews synthesize residue behavior, detection technologies, and toxicological considerations for this class, underscoring why benzimidazoles remain common analytes in multiresidue methods [17]. In multiresidue workflows, carbendazim is typically robustly quantified by LC–MS/MS; however, method scope should explicitly consider precursor pesticides (thiophanate-methyl) and conversion patterns during sample handling or metabolism. Field dissipation studies that report linked dynamics among thiophanate-methyl and carbendazim highlight this interconversion issue and support including both analytes where relevant [18].

Dithiocarbamates (e.g., mancozeb, metiram, thiram, ziram, propineb) are a long-used, multi-site fungicide group. They are analytically challenging because enforcement often relies on a common moiety approach (acid digestion to carbon disulfide, CS2), which does not uniquely identify the parent compound and can be confounded by natural CS2 background or matrix-derived interferences. Updated European laboratory guidance documents reflect continuing method refinements (e.g., hydrolysis conditions, partitioning, GC measurement) aimed at improving reliability and comparability of CS2-based results. Recent toxicology and methodology discussions emphasize that dithiocarbamates also form specific degradation products (e.g., ethylene thiourea, ETU; propylene thiourea, PTU) with their own hazard relevance, creating a strong rationale to expand monitoring beyond CS2 where feasible (e.g., targeted LC–MS/MS for ETU/PTU alongside CS2 for enforcement) [19]. Because SEA diets include many leafy vegetables and herbs (matrices prone to strong matrix effects), careful QA/QC (recoveries, precision, and measurement uncertainty) is essential when interpreting dithiocarbamate results in multiresidue contexts.

Emerging fungicides in residue analysis include newer modes of action with rising market penetration, compounds used in niche tropical crops, and metabolites/transformants increasingly recognized as toxicologically or regulatory relevant. Two practical trends are notable. First, laboratories are moving toward HRMS workflows that combine targeted quantification with suspect screening to detect unexpected residues and metabolites in complex foods [20]. Second, routine triple-quadrupole methods are expanding panels and using cross-platform confirmation (LC–MS/MS plus GC–MS/MS) to reduce false positives/negatives for multiclass residue enforcement [21]. In SEA monitoring specifically, market-basket style surveys in Vietnam [22] and Malaysia demonstrate that multi-residue detection is feasible with modified QuEChERS and LC–MS/MS, and they provide real-world evidence that fungicides appear alongside insecticides in frequently consumed vegetables supporting the need to keep fungicide panels broad and regularly updated [23]. Major fungicide chemical classes relevant to multiresidues analysis in SEA foods could be seen in Table 1. Despite extensive classification, translating fungicide diversity into robust multiresidue analytical methods remains challenging due to conflicting extraction and detection requirements across classes.

## 4. Microchemical Sample Preparation Strategies

### 4.1. Miniaturized QuEChERS Approaches

Microchemical sample preparation has emerged as a key strategy to overcome the limitations of conventional extraction methods, particularly in reducing solvent consumption, improving throughput, and enhancing compatibility with multiclass analyte systems. QuEChERS remains the dominant framework; however, its conventional format is increasingly limited for multiclass fungicide analysis due to matrix effects and insufficient selectivity Maintaining comparable solvent-to-sample ratios. Recent synthesis papers also highlight QuEChERS miniaturization as part of broader trends toward greener, higher-throughput, and more modular analytical pipelines [37]. Conventional QuEChERS often uses 10 g test portions and 10 mL acetonitrile (MeCN) with salt-driven phase separation and dispersive SPE (d-SPE) cleanup. Micro-QuEChERS scales these steps down typically to 0.2–2 g test portions and 0.5–4 mL MeCN using smaller centrifuge tubes (2–15 mL), shorter vortex/centrifugation times, and reduced d-SPE masses, while preserving the key physicochemical functions with efficient partitioning into MeCN, water removal via MgSO_4_, and selective removal of matrix co-extractives with sorbents. Maintaining solvent-to-sample ratios preserves extraction efficiency; however, method robustness depends strongly on matrix homogeneity and analyte distribution [38]. A key limitation is increased subsampling variability in miniaturized formats, which may compromise reproducibility in heterogeneous food matrices. For example, quality-control discussions emphasize that shrinking from 10 g to 1 g can increase variance unless comminution/homogenization is highly effective; thus, micro-QuEChERS works best when milling/homogenization is robust and when labs implement replicate strategies or improved mixing for representative aliquots [39].

Buffered QuEChERS (acetate or citrate) is often retained in miniaturized formats to stabilize base-/acid-labile fungicides and improve reproducibility across matrices; the main change is proportional reduction of salt masses and tube volume. Trends papers note that these “modular” adjustments help maintain ruggedness across commodity groups [37]. Micro-QuEChERS is often paired with direct LC–MS/MS injection after dilution/filtration, or with minimal evaporation (e.g., gentle N_2_ blowdown of ≤1–2 mL) to avoid losses of volatile/semi-volatile analytes and to speed throughput. High-throughput/green reviews increasingly position these “evaporation-light” workflows as preferable, particularly in routine monitoring labs [40]. Some recent green-analytical literature highlights coupling micro-QuEChERS extracts to microextraction/enrichment (e.g., DLLME-type steps) to improve sensitivity while keeping total solvent low. This strategy is mainly used when target residues are expected at very low µg/kg levels and matrix effects are severe [38]. Low-solvent QuEChERS variants reduce MeCN volume and sometimes replace part of the MeCN with water (for dry matrices) to facilitate partitioning. The direction is consistent with green analytical chemistry priorities: minimize hazardous solvent use and maximize sample throughput. Recent high-throughput reviews explicitly describe miniaturized QuEChERS as a pathway to cut solvent volumes while remaining compatible with LC–MS/MS screening.

For dry matrices (notably rice), low-solvent strategies commonly add a measured water volume before MeCN to promote hydration and reproducible extraction. A Vietnam-focused high-capacity method for rice illustrates this logic clearly with 2 g rice, add 5 mL water and 10 mL MeCN (with 1% acetic acid) prior to salting-out, then further cleanup and concentration before LC–MS/MS and GC–MS/MS analysis. While this example is not “micro-QuEChERS” in solvent volume, it demonstrates the hydration principle used in miniaturized rice workflows (water addition enables smaller test portions without losing extraction robustness) [41].

Sorbent optimization in miniaturized d-SPE including primary–secondary amine (PSA), octadecylsilane (C18), graphitized carbon black (GCB), and enhanced matrix removal–lipid (EMR-Lipid). Because micro-QuEChERS typically uses smaller extract volumes and sometimes higher matrix-to-solvent ratios, cleanup efficiency becomes even more important for controlling ion suppression/enhancement in LC–MS/MS. PSA is widely used to remove fatty acids, sugars, and some organic acids. It is especially helpful for fruit/vegetable matrices with high co-extracted polar interferences that otherwise elevate baseline noise or suppress ionization. C18 targets nonpolar interferences (lipids, waxes) and is often combined with PSA in produce matrices; however, excessive C18 can reduce recoveries for very nonpolar analytes [42]. GCB is effective for pigments (chlorophyll, carotenoids), but it can retain planar pesticides/metabolites, causing low recoveries if overused. This tradeoff is repeatedly noted in comparative cleanup studies [43]. Last, EMR-lipid is designed for stronger lipid removal than classical C18/PSA mixtures and has been evaluated across complex matrices. Food-analytics studies show it can reduce matrix effects and improve chromatographic cleanliness, although its cost and occasional analyte losses necessitate validation per matrix/analyte set [44].

### 4.2. Advanced Microextraction Techniques

Advanced microextraction techniques enhance selectivity, preconcentration, and matrix clean-up in multiclass fungicide residue analysis; however, their performance remains highly method-specific and cannot be generalized across all fungicide classes or food matrices [45]. In Southeast Asian food systems, these techniques are particularly relevant because high-water vegetables, pigment-rich commodities such as chilies and leafy greens, and complex matrices such as spices, oils, and tea can intensify matrix effects and compromise trace-level quantification. Therefore, advanced microextraction should be positioned as a targeted analytical strategy for improving sensitivity and matrix tolerance rather than as a universal replacement for QuEChERS-based workflows while) microextraction is particularly valuable to suppress matrix effects while meeting stringent default limits (commonly 0.01 mg/kg for many analyte commodity pairs in some regulatory settings) [8].

SPME is a solvent-minimized technique where analytes partition into a sorptive coating (fiber/Arrow/thin-film) from headspace (HS) or directly from liquid (DI-SPME). It is especially attractive for relatively volatile or semi-volatile fungicides (and some transformation products), and for GC-based workflows where HS-SPME can simultaneously provide clean-up and preconcentration [46]. Recent developments SPME Arrow and thin-film SPME expand surface area, improve robustness, and enhance sensitivity for multi-residue monitoring [47]. In Central Vietnam, market/harvest-time vegetables showed median individual pesticide residues of 0.007–0.037 mg/kg, while extreme cases reached 38.6 mg/kg total pesticide concentration in mustard greens and 32.1 mg/kg in green onions, indicating the need for methods that remain reliable across both trace-level and highly contaminated samples [8]. While QuEChERS–LC/GC–MS/MS remains common for such surveys, SPME-based screening (e.g., HS-SPME–GC–MS/MS for more GC-amenable fungicides) can be strategically deployed for rapid follow-up confirmation, hot-spot monitoring, and process-control checks (washing, blanching, cooking) with minimal solvent and reduced laboratory waste [47].

Dispersive liquid–liquid microextraction (DLLME) forms a transient dispersion of extraction solvent microdroplets in the aqueous phase (often assisted by disperser solvents, vortexing, ultrasound, or effervescence), enabling high enrichment factors in minutes [48]. For fungicides, DLLME is practical for aqueous food extracts (vegetable homogenate aqueous phase, fruit juices, herbal infusions) and can be paired with LC–MS/MS for polar to moderately nonpolar analytes. Method development continues to emphasize greener solvent systems, salt/ionic-strength tuning, and miniaturized phase-separation strategies compatible with routine labs [49]. In SEA food monitoring, DLLME coupled with LC–MS/MS or GC–MS has been used to quantify fungicides such as carbendazim, metalaxyl, tebuconazole, and azoxystrobin in rice, vegetables, and fruits. Reported residue concentrations typically range from 0.002–0.150 mg/kg in rice [50] and leafy vegetables from Vietnam, Thailand, and Malaysia, while higher levels up to 0.30 mg/kg have occasionally been detected in intensively cultivated vegetables such as mustard greens and choy sum. Method detection limits using DLLME-based protocols commonly reach 0.1–5 µg/kg with recoveries of 75–115%, demonstrating suitability for trace-level multiresidue analysis in regional food systems [28].

Hollow-fiber liquid-phase microextraction (HF-LPME) is a membrane-protected microextraction approach where analytes migrate through (or into) a supported liquid membrane held in a porous hollow fiber, offering strong clean-up and reduced matrix interferences often critical for pigments, waxes, and co-extractives typical of herbs, leafy greens, and chilies [51]. HF-LPME can be configured as two-phase or three-phase systems depending on analyte ionization and target polarity, making it adaptable to multiclass fungicides (e.g., azoles with basic character versus neutral QoI fungicides) [52]. A recent multiclass HF-LPME coupled to LC/MS illustrates how the technique supports simultaneous analysis of diverse pesticide classes with improved tolerance to complex matrices. In SEA monitoring scenarios where residue levels can span orders of magnitude, HF-LPME can be advantageous for confirmatory re-analysis of difficult matrices (herbs, leafy vegetables) that otherwise show severe ion suppression/enhancement in LC–MS/MS. Reported concentrations typically range from approximately 0.002–0.08 mg/kg in vegetables and 0.001–0.05 mg/kg in rice and fruit samples, generally below established maximum residue limits but indicating continuous agricultural exposure [53].

Magnetic solid-phase extraction (MSPE) employs functionalized magnetic sorbents (e.g., Fe_3_O_4_-based composites, MOFs/COFs, carbon materials, PSA-like functionalities) dispersed into the sample; after binding, a magnet isolates the sorbent for washing and elution. This format is fast, scalable, and effective for pigment-rich and high-water matrices because it integrates extraction and dispersive clean-up [54]. Recent work demonstrates MSPE for mixed panels including fungicides (e.g., automated MSPE for multiple fungicides in fruit matrices) [55]. MOF/zeolitic or carbon–magnetic hybrids have also been widely investigated as high-capacity sorbents in pesticide workflows, reflecting the broader trend toward higher selectivity and greener operation [56]. In Central Vietnam, fungicide-relevant targets such as difenoconazole were frequently detected (41% detection frequency among ten targets), with median residues within 0.007–0.037 mg/kg ranges exactly the concentration window where MSPE’s enrichment plus matrix clean-up can improve quantification robustness [8]. In Thailand, frequent detections and exceedances included carbendazim 1.5 mg/kg (papaya) and metalaxyl 0.11 mg/kg (cabbage), where MSPE can be used either as a stand-alone micro-cleanup/enrichment or as a selective secondary clean-up after a small-scale extraction [57].

Pipette-tip SPE (PT-SPE) miniaturizes classical SPE into disposable tips packed (or coated) with sorbent, enabling rapid “aspirate–dispense” cycles for extraction and clean-up using milliliter-to-submilliliter volumes. Tip formats support automation and parallelization (multi-channel pipettes/robots), making them attractive for monitoring programs handling hundreds of vegetable samples weekly [58]. Recent designs include engineered tip sorbents (e.g., biochar-derived materials and other high-surface-area phases) that broaden analyte coverage and improve sustainability [59]. Although some recent tip-SPE demonstrations focus on non-pesticide analytes, the same device logic (micro-scale sorbent bed with rapid mass transfer) is directly transferable to fungicide multi-residue panels in food extracts. Thai surveillance reported residues at or above default limits such as fipronil 0.02 mg/kg (several vegetables) and chlorfenapyr 0.13 mg/kg, indicating the practical need for methods that are both sensitive and field-lab friendly [57]. PT-SPE is a strong candidate for decentralized provincial labs because it reduces solvent, glassware, and operator time while maintaining compatibility with LC–MS/MS confirmatory analysis.

Stir bar sorptive extraction (SBSE) uses a coated magnetic stir bar (traditionally PDMS; increasingly MOF/COF and other advanced coatings) to sorb analytes while stirring, providing higher phase volume than SPME fibers and often higher sensitivity for hydrophobic fungicides [60]. SBSE is particularly useful for liquid matrices (tea infusions, fruit juices, extracted aqueous phases) and can be coupled to thermal desorption (GC) or solvent back-extraction (LC). A recent SBSE–HPLC–MS/MS multi-residue workflow (demonstrated for large pesticide panels) illustrates the scalability of sorptive approaches when high sensitivity is required [61]. Vietnam and Thailand datasets show that vegetables can contain residues from low µg/kg–tens of µg/kg medians up to mg/kg “hotspot” levels, which supports a tiered monitoring logic including routine high-throughput screening, sorptive enrichment (SBSE/SPME) for improved sensitivity or difficult matrices, and confirmatory LC–MS/MS with matrix-matched calibration [8,57].

### 4.3. Green and Sustainable Microextraction

Green and sustainable microextraction techniques have become increasingly important in modern food contaminant analysis, particularly in response to stricter environmental regulations and the need for sustainable laboratory practices. Traditional extraction methods such as liquid–liquid extraction and Soxhlet extraction often require large volumes of toxic organic solvents and generate substantial chemical waste. In contrast, microextraction approaches minimize solvent consumption, reduce energy use, and enable high analytical sensitivity for trace contaminants in complex food matrices. These techniques are particularly relevant for monitoring pesticide residues, fungicides, and other contaminants in Southeast Asian food systems, where agricultural intensification has increased the need for rapid and sustainable analytical methods [45]. Green microextraction strategies align with the principles of Green Analytical Chemistry (GAC), which emphasize reduced reagent consumption, minimal waste generation, safer chemicals, and energy efficiency. Modern microextraction approaches including deep eutectic solvent-based extraction, ionic liquid-based microextraction, ultrasound-assisted extraction, and solvent-free techniques have been widely adopted in food safety monitoring. These techniques not only improve analytical performance but also significantly reduce environmental impact compared with conventional extraction protocols [62].

Deep eutectic solvents (DES) have emerged as one of the most promising green alternatives to traditional organic solvents in microextraction procedures. DES are typically formed by combining hydrogen bond donors and acceptors, such as choline chloride with organic acids, sugars, or alcohols, resulting in a low-melting liquid mixture with tunable polarity and high solvation capacity. Their low toxicity, biodegradability, and ease of preparation make them particularly suitable for sustainable analytical applications [63]. In food contaminant analysis, DES-based microextraction techniques such as DLLME, LPME, and single-drop microextraction (SDME) have demonstrated excellent extraction efficiency for pesticides, fungicides, and heavy metals. For example, DES-SDME methods have been successfully applied for the determination of trace pesticides including metribuzin, dichlorvos, and fenthion, achieving detection limits in the low µg/kg range while using less than 100 µL of extraction solvent [64]. In SEA food systems, DES-based microextraction has been increasingly explored for monitoring pesticide residues in commodities such as rice, tropical fruits, and vegetables. Studies conducted on rice samples from Thailand and Vietnam reported recovery rates between 85–110% for organophosphate and pyrethroid pesticides using DES-DLLME combined with GC-MS. Similarly, DES-based extraction of fungicides in chili and leafy vegetables from Indonesia achieved limits of detection below 5 µg/kg while reducing solvent consumption by more than 90% compared with conventional liquid–liquid extraction. These results demonstrate the strong potential of DES systems in sustainable food monitoring programs across SEA [63].

Ionic liquids (ILs) represent another class of environmentally friendly solvents widely applied in microextraction techniques. These salts, which remain liquid at relatively low temperatures, possess unique physicochemical properties such as negligible vapor pressure, high thermal stability, and tunable polarity. Because of these characteristics, ILs are highly effective extraction media for trace organic contaminants in food samples [62]. Ionic liquid-based microextraction is commonly implemented through dispersive liquid–liquid microextraction (IL-DLLME), hollow-fiber microextraction, or ultrasound-assisted microextraction. These methods typically require only a few microliters of ionic liquid, enabling efficient enrichment of analytes while drastically reducing solvent usage. For instance, ultrasound-assisted IL-DLLME has been used for trace metal determination in food samples, demonstrating high extraction efficiency and rapid sample preparation [65].

Solvent-free microextraction techniques represent another important direction in green analytical chemistry. SPME and SBSE are among the most widely used solvent-free approaches for food contaminant analysis. These methods rely on polymer-coated fibers or bars that adsorb analytes directly from the sample matrix without requiring organic solvents [62]. Ultrasound-assisted microextraction (UAME) further enhances extraction efficiency by generating acoustic cavitation, which improves mass transfer between the sample matrix and extraction solvent. The formation and collapse of microscopic bubbles disrupt cell structures and accelerate analyte release, enabling rapid extraction within minutes [66].

The development of green microextraction techniques is guided by the twelve principles of GAC. These principles emphasize strategies such as minimizing sample size, reducing hazardous reagents, increasing automation, and integrating analytical processes to reduce energy consumption. Microextraction methods inherently comply with many of these principles because they require minimal solvent volumes, shorter extraction times, and smaller sample quantities [67]. For example, conventional liquid–liquid extraction of pesticide residues may require 50–100 mL of organic solvent per sample, whereas microextraction methods typically use less than 1 mL and often only a few microliters. This reduction significantly decreases chemical waste and laboratory costs while improving occupational safety. Furthermore, microextraction techniques are compatible with automated analytical platforms, enabling high-throughput food safety monitoring programs. To quantitatively evaluate the environmental performance of analytical methods, several greenness assessment tools have been developed, including the Analytical Eco-Scale and Analytical GREEnness (AGREE) metrics. These tools allow researchers to compare analytical procedures based on criteria such as solvent toxicity, energy consumption, waste generation, and occupational hazards [68]. From a critical perspective, the reviewed microchemical techniques show distinct but complementary performance profiles rather than a single linear improvement over conventional methods. Miniaturized QuEChERS offers the strongest balance between throughput, robustness, and routine compatibility with LC–MS/MS, but it remains vulnerable to subsampling error and matrix-dependent ion suppression in heterogeneous foods. DLLME provides superior enrichment and low solvent consumption, yet its performance depends strongly on solvent selection, ionic strength, and phase-separation efficiency. SPME and SBSE offer clear sustainability advantages because they minimize or eliminate organic solvent use, but their applicability is narrower for highly polar or thermolabile fungicides. HF-LPME and MSPE improve selectivity and matrix clean-up, particularly in pigment-rich vegetables and herbs, although they require greater optimization and may reduce routine throughput. DES-based extraction represents a promising green alternative, but broader regulatory adoption remains limited by method standardization and validation gaps. Therefore, the main trend is not the universal replacement of conventional extraction, but the emergence of matrix-adapted, hybrid workflows that balance sensitivity, selectivity, sustainability, and regulatory practicality.

### 4.4. Advanced Extraction Materials

Recent developments in sample preparation have introduced advanced extraction materials as next-generation sorbents to enhance selectivity, enrichment efficiency, and matrix clean-up in multiclass fungicide residue analysis. Among these materials, metal–organic frameworks (MOFs), covalent organic frameworks (COFs), and functional hybrid nanomaterials have gained significant attention due to their tunable pore structures, high surface area, and chemically adjustable adsorption sites [3]. MOFs are crystalline porous materials composed of metal ions or clusters coordinated with organic ligands, enabling highly selective interactions with target analytes. Their application in analytical chemistry has expanded rapidly, particularly in SPE, MSPE, SPME, and stir-bar sorptive extraction (SBSE) systems. These materials provide superior adsorption capacity and enhanced selectivity compared with conventional polymeric sorbents, especially for multiclass pesticide and fungicide residues in complex food matrices such as fruits, vegetables, tea, and cereals [69]. Recent advances also demonstrate the integration of MOF-decorated deep eutectic solvents (DES–MOFs) and magnetic MOF composites, which significantly improve extraction efficiency and reduce matrix interferences in LC–MS/MS and HRMS workflows. These hybrid systems combine the chemical tunability of MOFs with the environmental advantages of green solvents, making them highly relevant for sustainable analytical chemistry [70]. In addition, COF-based sorbents and graphene- or carbon-based hybrid nanostructures have emerged as promising alternatives due to their high chemical stability and π–π interaction capability, which is particularly effective for hydrophobic fungicides such as strobilurins and SDHIs. These materials are increasingly being incorporated into microextraction platforms to improve enrichment factors and lower detection limits in the ng/kg–µg/kg range [71]. Despite their advantages, challenges remain in terms of large-scale synthesis reproducibility, stability in complex food matrices, and standardization of analytical protocols, which currently limit routine regulatory adoption. Nevertheless, these advanced materials represent a key direction in the evolution of microchemical extraction strategies, particularly for high-throughput and sustainable pesticide residue monitoring. To facilitate method selection, Table 2. provides a comparative overview of reported recoveries (2019–2026), target matrices, analyte classes, and practical considerations.

## 5. Advances in Instrumental Detection

**Figure 3:**
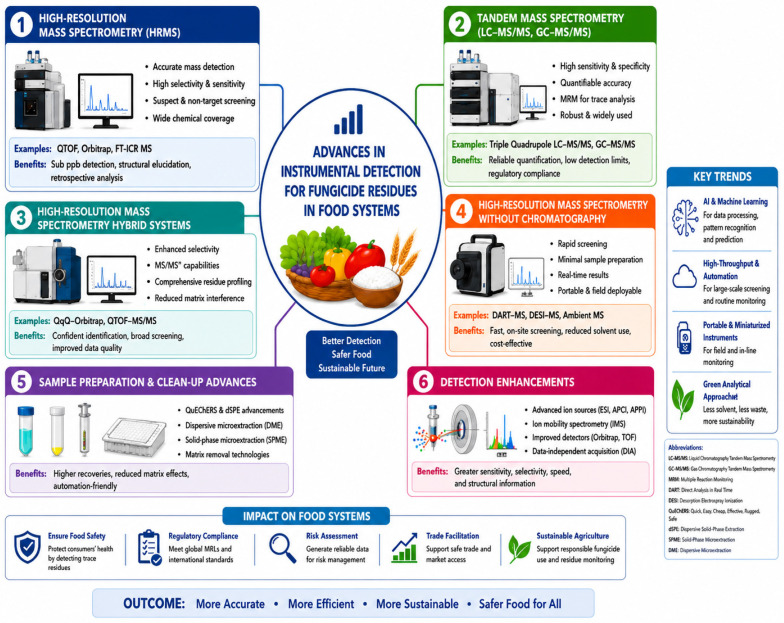
Advances in instrumental detection for fungicide residues in food systems.

A critical comparison of detection platforms indicates that instrumental advances should be interpreted according to analytical purpose rather than sensitivity alone. UHPLC–MS/MS remains the most suitable platform for routine targeted quantification because of its high sensitivity, reproducibility, and compatibility with regulatory validation. In contrast, HRMS platforms such as Orbitrap and QTOF provide stronger structural confirmation, suspect screening, and retrospective data analysis, but they require more complex data processing and may not always outperform triple-quadrupole MS in routine quantitative precision. GC–MS/MS remains important for volatile and thermally stable fungicides, whereas LC-based platforms are more appropriate for polar, thermolabile, and metabolite-related targets. Thus, the practical trend is toward complementary platform integration rather than technological substitution, where LC–MS/MS, GC–MS/MS, and HRMS are selected according to analyte chemistry, matrix complexity, and regulatory objective. Recent developments in analytical instrumentation have significantly enhanced detection capabilities; however, challenges related to matrix effects, co-elution, and multiclass complexity remain critical limitations. Modern workflows typically integrate optimized sample preparation methods such as QuEChERS or MSPE, followed by advanced chromatographic separation and mass spectrometric detection. High-performance techniques including UHPLC–MS/MS, GC–MS/MS, and HRMS platforms such as Orbitrap or time-of-flight (TOF) systems enable accurate identification and quantification of multiclass fungicides at trace levels (µg/kg to ng/kg). These technologies enable simultaneous monitoring of multiclass fungicides; however, co-elution and matrix effects remain significant analytical challenges, within a single analytical run. The integration of automated sample preparation, high-resolution detection, and advanced data processing algorithms has enhanced multiresidue screening capability, improved reproducibility, and facilitated regulatory monitoring of pesticide residues in global food supply chains. Advances in instrumental detection for fungicide residues in food systems could be seen in Figure 3.

### 5.1. UHPLC–MS/MS Developments

Ultra-high-performance liquid chromatography coupled with tandem mass spectrometry (UHPLC–MS/MS) has become the dominant platform for targeted multiclass fungicide analysis due to its high sensitivity and selectivity; however, its reliance on predefined analyte lists limits its applicability for emerging compounds and unknown metabolites. Over the past decade, advances in column technology, ionization interfaces, and mass spectrometric detection have significantly improved sensitivity, selectivity, and analytical throughput. Modern UHPLC systems improve separation efficiency; however, gains in speed and resolution may increase susceptibility to matrix-induced signal variability. When coupled with tandem mass spectrometry, UHPLC enables multi-residue detection of hundreds of analytes in a single run at sub-µg/kg levels, making it highly suitable for large-scale monitoring programs in SEA food systems where diverse commodities such as rice, tropical fruits, vegetables, spices, and seafood require comprehensive contaminant screening [73]. Recent instrumental innovations have focused on four major areas including triple quadrupole mass spectrometry platforms, scheduled multiple reaction monitoring (MRM) optimization, polarity switching strategies, and improved quantification approaches including matrix-matched calibration and isotope dilution. These developments collectively enhance analytical reliability when dealing with complex matrices typical of SEA foods, which often contain pigments, lipids, polyphenols, and essential oils that may cause ion suppression or enhancement during electrospray ionization [74].

Triple quadrupole (QqQ) mass spectrometers remain the most widely used detectors for quantitative multiresidue analysis in food safety laboratories. In a typical QqQ configuration, the first quadrupole (Q1) selects precursor ions, the second quadrupole (q2) serves as a collision cell for fragmentation, and the third quadrupole (Q3) monitors product ions. This tandem arrangement allows highly selective detection using specific precursor–product ion transitions, dramatically reducing background interference from complex food matrices [75]. Recent UHPLC–QqQ platforms incorporate faster scanning speeds and improved ion optics, enabling simultaneous monitoring of hundreds of pesticide transitions in a single analytical run. Such high-throughput capability is critical for multiresidue fungicide analysis in regulatory monitoring programs. For example, validated LC-MS/MS methods have been developed for simultaneous determination of fungicides such as azoxystrobin, boscalid, carbendazim, cyazofamid, prochloraz, and tebuconazole using optimized QuEChERS extraction and UHPLC separation. These methods typically achieve LOQ ≤ 10 µg/kg with recoveries between 70–120% and relative standard deviations below 20%, meeting international validation criteria for pesticide residue analysis [76]. Applications in SEA demonstrate the effectiveness of UHPLC–MS/MS for detecting fungicides in diverse commodities. Studies on tropical fruits such as mango, papaya, and longan have reported residues of triazole fungicides including tebuconazole and difenoconazole typically ranging from 0.005 to 0.2 mg/kg, while strobilurin fungicides such as azoxystrobin and pyraclostrobin are often detected at levels between 0.01 and 0.3 mg/kg depending on agricultural practices and pre-harvest intervals. Similar multiresidue surveys in rice and vegetables from Thailand, Vietnam, and Indonesia have demonstrated the capability of UHPLC–MS/MS systems to simultaneously quantify over 100 fungicides, including multiple fungicide classes, in a single run of less than 15 min.

One major advancement in tandem mass spectrometry acquisition strategies is the implementation of scheduled multiple reaction monitoring (sMRM). In conventional MRM methods, all transitions are monitored continuously throughout the chromatographic run, which can reduce dwell time and compromise sensitivity when a large number of analytes are included. Scheduled MRM addresses this limitation by monitoring each transition only within a defined retention time window corresponding to the expected chromatographic elution of the analyte. This targeted acquisition approach increases dwell time per transition and improves signal-to-noise ratios, thereby enhancing detection sensitivity for trace fungicide residues. Modern UHPLC–MS/MS platforms can monitor hundreds of transitions using sMRM while maintaining adequate cycle times and peak definition. The technique is particularly valuable for large multiresidue pesticide methods where more than 200 compounds are analyzed simultaneously [77]. In SEA monitoring programs, scheduled MRM has been widely applied to analyze fungicide residues in complex matrices such as spices, tea, and herbal products, which often contain co-extractives that interfere with detection. For instance, optimized sMRM methods have enabled reliable quantification of azole fungicides (e.g., propiconazole and epoxiconazole) in spice matrices at concentrations below 0.01 mg/kg. Such analytical sensitivity is essential for ensuring compliance with MRLs established by regulatory authorities including Codex Alimentarius and the European Union.

One of the most significant challenges in LC-MS/MS pesticide analysis is the matrix effect, which arises when co-extracted compounds suppress or enhance ionization efficiency in the mass spectrometer. Complex food matrices such as spices, tea, fermented foods, and tropical fruits often contain pigments, lipids, and phenolic compounds that can significantly influence analytical accuracy. Matrix effects can lead to biased quantification if not properly corrected [78]. Matrix-matched calibration is one widely adopted strategy to compensate for such effects. In this approach, calibration standards are prepared in blank matrix extracts rather than pure solvent, ensuring that both calibration standards and sample extracts experience similar ionization conditions during analysis. Studies have shown that matrix-matched calibration significantly improves quantitative accuracy in LC-MS/MS fungicides analysis compared with solvent-based calibration. An even more robust approach involves isotope dilution mass spectrometry (IDMS), in which isotopically labeled internal standards (e.g., deuterated or ^13^C-labeled analogues of fungicides) are added to samples prior to analysis. Because the labeled standard behaves identically to the target analyte during extraction, chromatographic separation, and ionization, it effectively corrects for matrix effects, recovery losses, and instrumental variability. This strategy is increasingly used in advanced food safety laboratories for high-accuracy quantification of pesticide residues [79]. In SEA studies, matrix-matched calibration and isotope dilution approaches have been successfully applied to quantify fungicide residues in commodities such as chili peppers, rice, and leafy vegetables. For example, monitoring programs in Thailand and Vietnam have reported azoxystrobin residues in chili peppers at levels between 0.02 and 0.15 mg/kg and tebuconazole residues in rice between 0.005 and 0.05 mg/kg using isotope-corrected UHPLC–MS/MS methods. These concentrations are typically below international MRL thresholds but highlight the importance of sensitive analytical techniques for food safety surveillance.

### 5.2. HRMS

HRMS provides powerful capabilities for suspect and non-target screening; however, limitations in quantitative robustness and data processing complexity restrict routine application. The increasing diversity of agrochemicals and their transformation products in modern agricultural systems particularly in intensively cultivated regions such as SEA requires analytical techniques capable of detecting hundreds of compounds simultaneously at trace concentrations. HRMS platforms, especially Orbitrap and quadrupole time-of-flight (Q-TOF) instruments coupled with liquid chromatography (LC-HRMS), provide high mass accuracy (<5 ppm), resolving power exceeding 50,000, and full-scan acquisition modes that enable both targeted quantification and broad non-target screening of contaminants in food systems [80]. Unlike conventional LC-MS/MS, which is optimized for predefined analytes, HRMS allows simultaneous detection of thousands of molecular features through full-scan data acquisition. This capability is particularly valuable for monitoring fungicides and their metabolites in agricultural commodities such as rice, fruits, vegetables, and spices commonly produced in Southeast Asia. HRMS methods can achieve detection limits at sub-µg/kg levels while maintaining high selectivity even in complex matrices due to precise mass measurement and isotopic pattern recognition.

Among HRMS technologies, Orbitrap and Q-TOF mass spectrometers are the most widely used platforms for pesticide and fungicide residue analysis. Orbitrap-based systems provide extremely high mass resolution and accurate mass measurement, making them particularly suitable for multi-residue screening and confirmation of unknown compounds. Q-TOF instruments, on the other hand, combine high-resolution mass measurement with rapid spectral acquisition, enabling efficient detection of multiclass pesticides in complex matrices such as fruits, vegetables, cereals, and herbal products. Applications of HRMS have been widely reported in agricultural commodities typical of SEA [81]. For example, analyses of rice, mango, chili, tea, and leafy vegetables have identified fungicides such as azoxystrobin, tebuconazole, carbendazim, and difenoconazole at concentrations typically ranging from 0.001 to 0.2 mg/kg, with occasional exceedances reported above 0.5 mg/kg in intensively treated crops. These residues are frequently detected in surveillance programs across countries such as Vietnam, Thailand, Indonesia, and Malaysia, reflecting the widespread use of fungicides for controlling fungal diseases in humid tropical climates.

Non-target screening (NTS) expands analytical scope but introduces significant challenges in data interpretation, identification confidence, and standard availability. Features detected in the chromatogram are filtered based on mass accuracy, isotopic pattern, retention time, and fragmentation spectra to identify potential contaminants. This strategy has proven particularly useful for discovering emerging fungicide metabolites formed through environmental transformation or food processing [82]. By reprocessing archived HRMS datasets, researchers can track historical contamination trends and identify previously overlooked compounds. For example, retrospective screening of archived fruit and vegetable samples using HRMS has revealed the presence of newly regulated fungicides such as fluxapyroxad and benzovindiflupyr that were not included in earlier targeted methods. This capability is particularly valuable in SEA, where agricultural practices evolve rapidly due to changing pest pressures and regulatory frameworks. Retrospective HRMS analysis has been used to reassess archived rice and tropical fruit samples, revealing historical contamination patterns for fungicides including azoxystrobin (0.01–0.12 mg/kg), carbendazim (0.02–0.3 mg/kg), and propiconazole (0.005–0.09 mg/kg). Such data provide critical information for risk assessment and regulatory decision-making. Although HRMS enables suspect and non-target screening, challenges related to data processing complexity, standard availability, and quantitative robustness limit its routine application in regulatory monitoring.

### 5.3. GC–MS/MS for Volatile Fungicides

Recent developments in GC–MS/MS instrumentation have significantly improved analytical performance for multiresidue pesticide monitoring. Modern triple quadrupole mass spectrometers operating in MRM mode provide enhanced selectivity and low detection limits, often reaching sub-microgram per kilogram levels. This capability is critical in SEA food systems where intensive agriculture and high humidity favor fungal diseases, resulting in extensive fungicide application on commodities such as rice, tropical fruits, and vegetables [83]. Monitoring studies in the region frequently report residues of fungicides such as tebuconazole, difenoconazole, propiconazole, azoxystrobin, and carbendazim in vegetables, fruits, and cereal products. For instance, multiresidue analyses have detected triazole fungicides including tebuconazole and difenoconazole at concentrations ranging from approximately 0.01 to 0.20 mg/kg in leafy vegetables and tropical fruits, while strobilurin fungicides such as azoxystrobin are often reported at 0.005–0.15 mg/kg in fruit commodities [84]. In certain cases, higher residues approaching 0.3 mg/kg have been observed in intensively treated crops, although most results remain below established MRLs [85].

Derivatization plays an essential role in GC–MS/MS analysis when target fungicides exhibit poor volatility, thermal instability, or insufficient chromatographic behavior. Chemical derivatization modifies functional groups within pesticides molecules, converting them into more volatile and thermally stable derivatives that are compatible with gas chromatographic separation. Common derivatization reagents include silylation agents (e.g., N-methyl-N-trimethylsilyl-trifluoroacetamide, MSTFA), acylation reagents, and alkylation agents. These reagents react with hydroxyl, carboxyl, or amine groups to improve volatility and peak symmetry during GC separation. In fungicide residue analysis, derivatization is particularly useful for compounds such as dithiocarbamates and certain polar metabolites of azole fungicides. Dithiocarbamates are typically converted to carbon disulfide (CS2) during acid digestion prior to GC analysis, enabling indirect quantification using GC–MS detection. This strategy has been widely used in monitoring programs because the parent compounds are unstable and difficult to analyze directly [86]. For triazole fungicides such as tebuconazole and propiconazole, derivatization may enhance chromatographic response and reduce matrix effects in complex food matrices like rice or fermented foods. Silylation reactions are commonly applied to improve detectability of hydroxyl-containing metabolites formed during degradation or food processing. These derivatized compounds produce characteristic fragmentation patterns in MS/MS spectra, facilitating accurate identification even at trace concentrations [82]. Comparative overview of recent instrumental approaches for fungicide-residue detection in SEA food matrices could be seen in Table 3.

## 6. Comparison Between Microchemical and Conventional Methods

A fundamental distinction between conventional and microchemical approaches lies in analytical efficiency, particularly in solvent consumption, extraction selectivity, and compatibility with high-throughput workflows. Traditional extraction methods often require tens to hundreds of milliliters of organic solvents such as acetonitrile, dichloromethane, or hexane to isolate pesticide residues from complex food matrices. These high solvent requirements not only increase analytical costs but also generate hazardous chemical waste, conflicting with modern principles of green analytical chemistry. In contrast, microchemical approaches employ miniaturized extraction systems that drastically reduce solvent consumption. For example, microextraction techniques such as SPME can operate with minimal or even solvent-free extraction phases, while modified QuEChERS methods typically reduce solvent usage by more than half compared with classical multiresidue extraction protocols. This reduction in solvent demand contributes to lower environmental impact and improved sustainability of fungicide monitoring programs [5]. Sample size requirements also differ substantially between these analytical strategies. Conventional extraction procedures generally require relatively large sample masses, often between 10–50 g of homogenized food material, to achieve reliable detection of trace pesticide residues. Microchemical approaches, however, employ miniaturized extraction formats capable of analyzing significantly smaller sample volumes or masses while maintaining adequate detection limits. Techniques such as DLLME, SPME, and single-drop microextraction rely on micro-scale extraction interfaces that allow rapid enrichment of analytes from small sample volumes. These miniaturized systems enhance extraction efficiency and facilitate analysis of limited or valuable samples, such as specialty foods or environmental monitoring samples [47].

Microchemical approaches improve throughput; however, increased speed may compromise reproducibility and method robustness if not carefully optimized. Conventional sample preparation procedures often involve multiple steps including solvent extraction, filtration, concentration, and clean-up, which collectively increase analysis time and reduce laboratory productivity. In contrast, microextraction-based techniques are designed to simplify sample preparation and reduce the number of processing steps. For example, the QuEChERS method integrates extraction and clean-up in a streamlined workflow that enables rapid multiresidue pesticide screening in fruits, vegetables, and processed foods [37]. As a result, microchemical approaches allow laboratories to process large numbers of samples within shorter timeframes, making them particularly suitable for routine monitoring of fungicide residues in regulatory surveillance programs [94]. Sensitivity is another critical parameter influencing analytical performance. Advances in microextraction technologies combined with modern chromatographic and mass spectrometric detection systems have significantly improved the sensitivity of fungicide residue analysis. Microchemical approaches often provide enhanced sensitivity due to analyte preconcentration; however, this advantage depends strongly on method optimization and matrix characteristics. For instance, microextraction techniques enhance analyte preconcentration prior to instrumental analysis, enabling trace-level detection of pesticide residues in complex matrices [95]. Enhanced sensitivity supports regulatory compliance; however, it depends strongly on matrix effects and calibration strategy. Despite these advantages, critical evaluation of microchemical approaches reveals several practical considerations. In terms of cost-effectiveness, microextraction techniques reduce solvent consumption and laboratory waste, which lowers operational costs over time. However, some specialized extraction devices or sorbent materials may require initial investment. Regarding scalability, many microchemical methods such as QuEChERS are already widely implemented in regulatory laboratories because they are relatively simple, reproducible, and compatible with high-throughput analytical platforms. Nonetheless, certain microextraction techniques requiring specialized instrumentation may be less accessible in routine laboratories [96]. Finally, the suitability of microchemical methods for developing countries is particularly promising. Their reduced reagent requirements, simplified workflows, and compatibility with portable analytical technologies make them attractive options for expanding pesticide residue monitoring capacity in resource-limited settings. Overall, microchemical approaches represent a significant advancement over conventional analytical techniques for fungicide residue determination, offering improved sustainability, efficiency, and analytical performance in modern food safety monitoring systems [97]. In addition to sustainability and analytical performance, the suitability of each microchemical preparation strategy depends strongly on its compatibility with instrumental detection platforms and its potential for automation or online coupling. Miniaturized QuEChERS and magnetic solid-phase extraction are more suitable for high-throughput liquid chromatography tandem mass spectrometry workflows, whereas solid-phase microextraction and stir-bar sorptive extraction offer stronger potential for automated or online coupling with gas chromatography or liquid chromatography systems. Dispersive liquid–liquid microextraction and hollow-fiber liquid-phase microextraction provide effective enrichment and matrix clean-up, but they require careful optimization before routine automation because phase separation, membrane stability, and equilibration time can affect reproducibility. To strengthen methodological interpretability, a direct comparison between conventional and microchemical extraction approaches is provided in Table 4. This comparison highlights fundamental differences in solvent consumption, sample requirements, extraction time, analytical sensitivity, recovery performance, throughput, cost efficiency, and environmental impact. Conventional extraction techniques such as LLE, SPE, and Soxhlet generally require large solvent volumes and longer processing times, which limit their sustainability and throughput. In contrast, microchemical approaches including miniaturized QuEChERS, DLLME, SPME, MSPE, and DES-based extraction significantly reduce solvent usage while improving or maintaining analytical sensitivity when coupled with LC–MS/MS or HRMS systems. These improvements are consistent with the principles of Green Analytical Chemistry and demonstrate the transition toward more sustainable, high-throughput analytical workflows in multiclass fungicide monitoring.

## 7. Current Challenges, Analytical Gaps, and Future Perspectives

Despite significant advances, major analytical challenges persist, particularly in the determination of highly polar fungicides, transformation products, and matrix-bound residues. One major limitation involves the determination of highly polar fungicides, which exhibit physicochemical properties such as low partition coefficients and high water solubility that complicate extraction and chromatographic retention. Conventional multiresidue approaches such as QuEChERS combined with LC–MS/MS are optimized for moderately polar compounds, but they often show poor recovery and retention for highly polar fungicides and their metabolites. Consequently, dedicated analytical workflows or specialized stationary phases are often required, reducing analytical throughput and limiting the scope of multiclass monitoring programs. These limitations highlight a critical gap in current multiresidue methods, particularly for integrating highly polar compounds into routine workflows [80]. Another significant challenge concerns the occurrence of conjugated and bound residues, which are formed when fungicides undergo metabolic transformation in plants, microorganisms, or food processing environments. These residues may exist as glycosides, sulfates, or other conjugated derivatives that are not directly detectable by conventional analytical methods targeting parent compounds. As a result, standard residue analysis may underestimate the total fungicide burden present in food matrices. The analytical characterization of such transformation products requires advanced sample preparation approaches, enzymatic hydrolysis, or high-resolution mass spectrometry techniques capable of identifying unknown metabolites. Furthermore, the toxicological significance of many conjugated residues remains insufficiently understood, creating additional uncertainty in risk assessment and regulatory evaluation.

The limited availability of certified reference standards for metabolites and emerging fungicides represents a critical bottleneck for accurate quantification and method validation. Many metabolites, degradation products, and newly developed fungicidal compounds lack commercially available analytical standards, making accurate quantification difficult. Without appropriate standards, analysts often rely on semi-quantitative approaches or surrogate calibration strategies that may introduce uncertainty into residue measurements. This issue is particularly relevant for emerging fungicides introduced to replace older active ingredients with unfavorable environmental profiles. The rapid evolution of agrochemical formulations has therefore outpaced the development of validated analytical standards, creating discrepancies between agricultural practice and monitoring capabilities [1]. The emergence of new fungicides and transformation products not yet incorporated into multiclass analytical methods further complicates monitoring efforts. Modern fungicide classes including novel SDHIs, oxysterol-binding protein inhibitors, and other recently introduced compounds may not be included in existing residue screening panels. Because regulatory monitoring methods are often validated for a predefined list of target analytes, newly registered fungicides may remain undetected until analytical methods are updated. This lag between pesticide innovation and analytical adaptation poses a risk for food safety monitoring programs and highlights the importance of high-resolution mass spectrometry and suspect screening workflows capable of detecting previously unrecognized residues [86]. A further analytical challenge involves severe matrix-induced ion suppression or enhancement during LC–MS/MS analysis. Food matrices such as fruits, vegetables, grains, and processed products contain numerous co-extractives including lipids, pigments, sugars, and organic acids that may interfere with electrospray ionization. These matrix effects can reduce signal intensity or produce variable responses, leading to inaccurate quantification of fungicide residues. Although cleanup strategies such as dispersive solid-phase extraction and matrix-matched calibration are commonly used to mitigate these effects, complete elimination remains difficult, particularly in complex matrices. Consequently, improving sample preparation strategies and developing more robust ionization techniques remain important research priorities in residue analysis [103]. Finally, biosensor-based screening methods, which are increasingly proposed as rapid alternatives to chromatographic techniques, face challenges related to cross-reactivity and selectivity. Many immunoassays and aptamer-based sensors rely on molecular recognition elements that may interact with structurally similar pesticides or metabolites, producing false-positive or false-negative results. While biosensors offer advantages such as portability, rapid detection, and potential field deployment, their analytical specificity is often lower than that of chromatographic-mass spectrometric approaches. Cross-reactivity therefore remains a critical limitation that must be addressed through improved sensor design, advanced nanomaterials, and integrated confirmatory analytical techniques [104].

Future analytical strategies should prioritize the integration of high-resolution mass spectrometry, AI-assisted data processing, and green microextraction techniques to address current limitations in multiclass residue analysis. One promising direction is the development of microfluidic-based extraction platforms, which enable miniaturized, rapid, and highly efficient sample preparation using extremely small volumes of solvents and samples. Microfluidic systems integrate extraction, separation, and detection steps within a single chip, thereby significantly reducing analysis time and reagent consumption compared with conventional extraction techniques such as liquid–liquid extraction or solid-phase extraction. Recent studies demonstrate that microfluidic devices coupled with fluorescence, electrochemical, or colorimetric detection provide sensitive detection of pesticide and fungicide residues in complex food matrices, making them suitable for high-throughput monitoring in food safety laboratories [105]. Nanomaterial-based sorbents show promising selectivity; however, reproducibility, cost, and scalability remain significant barriers to routine implementation. Nanomaterials such as graphene derivatives, carbon nanotubes, metal–organic frameworks, magnetic nanoparticles, and molecularly imprinted polymers have shown exceptional adsorption capacity and selectivity for pesticide molecules. When incorporated into extraction techniques such as modified QuEChERS or solid-phase extraction, these materials improve analyte enrichment, matrix cleanup efficiency, and detection sensitivity. Recent research also highlights the use of magnetic nanomaterials that allow rapid phase separation and facilitate automated workflows for multi-residue pesticide screening [106]. The increasing complexity of pesticide mixtures in food matrices has also stimulated the development of artificial intelligence (AI)-assisted non-target screening approaches. Non-targeted analysis using high-resolution mass spectrometry generates vast spectral datasets that are difficult to interpret using conventional workflows. Machine learning algorithms and advanced data-mining techniques can facilitate automated peak recognition, spectral deconvolution, and identification of unknown pesticide metabolites or transformation products. AI-assisted approaches offer potential for data interpretation; however, their reliability depends on training data quality and standardization of analytical workflows [107]. Finally, future research should align with the principles of sustainable analytical chemistry, particularly within SEA food systems where agricultural intensification and diverse commodity matrices pose unique analytical challenges. Sustainable analytical frameworks emphasize green sample preparation methods, reduced solvent consumption, energy-efficient instrumentation, and the use of biodegradable or reusable materials in analytical workflows. Integrating microfluidics, nanotechnology, and automated digital platforms can significantly reduce environmental impacts while maintaining analytical sensitivity and reliability. Such approaches are essential for establishing long-term monitoring strategies that support food safety, environmental protection, and sustainable agricultural development across SEA [108]. A key limitation in current literature is the lack of standardized evaluation criteria for comparing microchemical extraction techniques across different matrices and fungicide classes. Future research should adopt harmonized validation protocols and incorporate greenness assessment metrics to enable objective comparison and method selection.

## 8. Conclusions

Microchemical analytical techniques for multiclass fungicide residue determination demonstrate clear advancement in sensitivity, sustainability, and throughput; however, their applicability remains strongly matrix-dependent due to persistent matrix effects, variable extraction efficiency across fungicide classes, and inconsistent validation performance among food commodities. Although these methods are increasingly aligned with green analytical chemistry principles and show strong potential for routine food safety monitoring, their regulatory adoption is still constrained by limited harmonized validation protocols and insufficient cross-matrix comparative evidence. Therefore, future implementation in official monitoring systems will require standardized validation frameworks, inter-laboratory comparability, and stronger head-to-head evaluations against conventional reference methods to ensure robustness, reproducibility, and regulatory compliance.

## Figures and Tables

**Table 1:** Major chemical classes of fungicides frequently detected in SEA foods.

Fungicide Class	Representative Actives Commonly Relevant to SEA Foods (Examples)	Typical SEA Foods/Commodities Where Residues are Often Reported	SEA Region Signals (Examples of Monitoring Contexts)	Indicative Polarity (logP Range; Qualitative)	Example MRLs (EU Examples from 2019–2026 Literature/Regulatory Science)	Ref.
Azoles (DMI; triazoles/imidazoles)	Tebuconazole, Difenoconazole	Leafy greens, chili, rice, mango [24]	Vietnam, Malaysia export	3.5–4.5	Difenoconazole (wheat/rye grain): proposed 0.3 mg/kg	[25,26]
Strobilurins (QoI inhibitors)	Azoxystrobin, Pyraclostrobin	Fruits, vegetables, rice	Regional multiresidue	3.5–6.0	Azoxystrobin (import tolerance examples exist for tropical fruits such as mango/oil palm fruit in EFSA assessments; commodity-specific values apply)	[24,27,28]
SDHI fungicides	Boscalid, Fluopyram, Benzovindiflupyr	Vegetables, fruits	Intensive horticulture	3.5–5.5	Boscalid (pomegranates): proposed MRL increase 0.01 into 2.0 mg/kg. Fluopyram (broccoli): proposed 0.4 mg/kg.	[28,29,30]
Benzimidazoles	Carbendazim, Thiophanate-methy	Vegetables, spices	Legacy and enforcement monitoring	1.0–2.0	EU risk management for benzimidazoles includes updated EFSA considerations (import tolerances/legacy residues; commodity-specific)	[24,31,32]
Dithiocarbamates	Mancozeb	Tropical fruits, leafy greens	Plantation systems SEA	variable	EFSA has multiple recent MRL assessments for dithiocarbamates; example EFSA documentation discusses MRL setting/updates and scenarios	[33,34,35]
Emerging fungicides & metabolites	Cyazofamid and metabolite CCIM	Vegetables, specialty crops	HRMS-based surveillance	variable	EU regulatory lists show ongoing MRL updates for multiple newer actives (commodity-specific amendments in recent OJ/EU regulations)	[36]

**Table 2:** Comparative Overview of Microchemical Extraction Strategies for Multiclass Fungicide Analysis in Food Matrices.

Extraction Method	Typical Matrices	Fungicide Classes	Reported Recovery (%)	Advantages	Limitations	Key Analytical Implication	Ref.
Miniaturized QuEChERS	Leafy greens, chili, rice, tropical fruits	Azoles, QoIs, SDHIs, benzimidazoles	70–120 (leafy vegetables: azoles/SDHIs, LC–MS/MS validated multi-residue methods in peer-reviewed pesticide journals)	High throughput	Subsampling error	Routine regulatory workflow	[38,40,41]
DLLME	Vegetable aqueous extracts, fruit juices	Carbendazim, tebuconazole, azoxystrobin	75–115 (rice/vegetable matrices; LC–MS/MS validation studies in multiresidue pesticide analysis literature)	High enrichment	Solvent optimization sensitive	High preconcentration capability	[28,49,50]
SPME	Leafy greens, herbs, high-pigment vegetables	Volatile/semi-volatile fungicides	70–110 (HS-SPME–GC-MS/MS validated for pesticide residues in vegetables and herbs)	Solvent-free	Limited polarity range	Green analytical method	[8,46,47]
HF-LPME	Vegetables, rice, fruit samples	Azoles, QoIs, SDHIs	80–110 (LC–MS/MS validation in complex vegetable matrices, peer-reviewed microextraction studies)	Strong cleanup	Lower throughput	High selectivity for complex matrices	[51,52,53]
MSPE	Pigment-rich fruits and vegetables	Difenoconazole, carbendazim, azoxystrobin	85–110 (magnetic sorbent LC–MS/MS validation in fruit/vegetable pesticide studies)	Fast & scalable	Sorbent cost	Reduces ion suppression	[54,55,57]
DES-Based Extraction	Rice, chili, leafy vegetables	Azoles, organophosphates, pyrethroids	85–110 (green solvent extraction validated in LC–MS/MS pesticide residue studies)	Green chemistry	Limited standardization	Sustainable extraction	[63,64]
MOF/COF-based SPE & MSPE (Advanced materials)	Complex fruits, tea, spices	Multiclass fungicides incl. emerging residues	80–120 (HRMS/LC-MS/MS validation in advanced sorbent studies)	High selectivity	Synthesis complexity	Next-gen sorbents	[72]

**Table 3:** Comparative Analytical Performance of Instrumental Approaches for Multiclass Fungicide Residue Analysis.

Instrument Approach	Best Suited Fungicide Classes/Compounds	LOQ/LOD (Typical Reported Range)	Main Strengths	Main Limitations	Typical Analytical/Regulatory Usefulness	Representative SEA Food Examples	Ref.
UHPLC–MS/MS (Triple Quadrupole, MRM)	Azoles (difenoconazole, tebuconazole), SDHIs, strobilurins, benzimidazoles	LOD: 0.001–0.01 mg/kg; LOQ: 0.005–0.01 mg/kg (leafy vegetables, rice, fruit matrices; validated EFSA SANTE-compliant methods)	Highest sensitivity, high selectivity, robust for routine monitoring	Matrix effects (ion suppression/enhancement), requires isotopic standard	Gold standard for regulatory enforcement (EU SANTE compliant)	Chili, leafy vegetables, mango, rice (Vietnam, Malaysia, Thailand)	[87,88]
GC–MS/MS	Volatile/semi-volatile fungicides (captan, chlorothalonil, dithiocarbamate derivatives)	LOQ: 0.005–0.05 mg/kg (spices, tea, vegetables; derivatized methods)	High confirmation power, strong spectral library match	Requires derivatization; not suitable for polar fungicides	Confirmatory analysis for GC-amenable compounds	Tea, spices, kale, long bean (SEA vegetables)	[4]
LC–HRMS (Orbitrap/QTOF)	Broad fungicide classes + metabolites/transformation products	Targeted LOQ: 0.005–0.05 mg/kg (rice/vegetables); suspect screening: qualitative detection only (matrix dependent)	Non-target capability, metabolite detection, retrospective analysis	High cost, complex data processing	Surveillance, suspect & non-target screening	Mixed vegetables, export fruits (SEA monitoring programs)	[1]
HPLC-UV/DAD (legacy method)	Single-class fungicides (benzimidazoles, older compounds)	LOQ: 0.05–0.5 mg/kg (clean matrices only, e.g., cereals)	Low cost, simple instrumentation	Low selectivity, unsuitable for multiclass trace analysis	Screening only (non-enforcement grade)	Limited use in local labs	[7]
QuEChERS + LC–MS/MS (hybrid workflow)	Multiclass fungicides (azoles, SDHIs, QoIs)	LOQ: 0.001–0.01 mg/kg (vegetables/rice; EU SANTE validation)	Fast, scalable, cost-efficient	Subsampling error in heterogeneous matrices	Routine national monitoring	Chili, leafy vegetables, rice (SEA)	[3]
LC–Orbitrap HRMS	Multiclass fungicides: azoles, SDHIs, strobilurins, benzimidazoles + metabolites	LOQ: 0.005–0.02 mg/kg (leafy vegetables, fruits; matrix dependent)	Full-scan acquisition, accurate mass (<5 ppm), retrospective analysis, suspect screening	High cost, complex data processing, lower routine throughput than QqQ	Confirmatory and non-target screening for emerging fungicides and metabolites	Mango, chili, leafy vegetables, rice (SEA monitoring studies)	[89]
LC–QTOF-MS/MS (Quadrupole Time-of-Flight HRMS)	Broad fungicide panels incl. SDHIs, azoles, QoIs, transformation products	LOQ: 0.005–0.01 mg/kg (rice, vegetables; LC-HRMS validated workflows)	Accurate mass fragmentation, library matching, multi-residue profiling	Slightly less sensitive than QqQ in routine MRM quantification	Regulatory screening and confirmation of unknown residues	Rice, soybean, peanut, vegetables	[90]
GC–Orbitrap HRMS (GC–EI high-resolution MS)	GC-amenable fungicides: chlorothalonil, captan, folpet, dithiocarbamate derivatives (indirect)	LOQ: 0.005–0.02 mg/kg (tea, spices; derivatized matrices)	Ultra-high selectivity (≤5 ppm), EI spectral compatibility with libraries (NIST), broad screening scope	Requires derivatization for polar analytes; not suitable for thermolabile compounds	High-confidence confirmatory analysis and retrospective screening	Tea, spices, chili, leafy vegetables	[91]
LC–IM–QTOF-MS (Ion Mobility–HRMS 3D separation)	Complex-matrix fungicides incl. azoles, SDHIs, and co-eluting metabolites	LOQ: 0.01–0.05 mg/kg (targeted mode); improved qualitative detection	Adds collision cross section (CCS), reduces isobaric interference, improves confidence	Limited CCS libraries; emerging validation standards	Advanced confirmatory screening in highly complex matrices	Herbal products, spices, chili, tea	[92]
Ambient MS (DART–HRMS/DESI–MS)	Rapid screening of surface fungicide residues (semi-volatile/polar compounds)	LOQ: 0.04–0.2 mg/kg (typical reported range)	Minimal sample prep, ultra-fast analysis (seconds–minutes), high throughput	Poor matrix robustness, limited quantitative accuracy	Field screening/rapid surveillance prior to confirmatory LC–MS/MS	Fresh fruits, vegetables, market samples	[93]

**Table 4:** Comparative Evaluation of Conventional and Microchemical Extraction Methods for Multiclass Fungicide Residue Analysis.

Parameter	Conventional Methods (LLE/SPE/Soxhlet/Classical QuEChERS)	Microchemical Methods (Mini-QuEChERS/DLLME/SPME/MSPE/DES-Based)	Key Analytical Implication	Ref.
Solvent consumption	High (10–150 mL per sample)	Very low (µL–≤5 mL per sample)	Major reduction in hazardous waste (>80–95%), aligns with Green Analytical Chemistry (GAC)	[98,99]
Sample size requiremen	Large (10–500 g)	Small (0.2–5 g)	Enables field-scale monitoring and limited sample availability analysis	[100]
Extraction time	Long (1–6 h)	Short (2–30 min)	Increases throughput and reduces labor intensity	[72]
LOD/LOQ performance	Moderate (LOD: 0.01–0.1 mg/kg; LOQ: 0.05–0.5 mg/kg)	High sensitivity (LOD: ng/kg–µg/kg; LOQ: 0.001–0.02 mg/kg with LC–MS/MS or HRMS)	Microextraction improves enrichment factor and instrumental sensitivity	[3]
Recovery (%)	70–110% (variable across matrices)	75–120% (more stable after optimization)	Improved reproducibility due to enhanced selectivity and reduced matrix load	[101]
Throughput	Low–moderate	High (parallel/miniaturized/automatable)	Suitable for large-scale surveillance programs	[3]
Operational cost	High (solvent, energy, and consumables)	Low–moderate	Reduced per-sample cost in routine monitoring	[72]
Environmental footprint	High (chemical waste intensive)	Low (green solvents/solvent-free options)	Strong compliance with sustainability metrics (AGREE/Eco-Scale)	[102]
Matrix effect control	Often requires extensive clean-up (d-SPE/SPE)	Improved suppression control (MSPE/SPME/DES reduce co-extractives)	Better LC–MS/MS signal stability in complex SEA matrices	[3]
Instrument compatibility	LC–MS/MS, GC–MS (after cleanup)	UHPLC–MS/MS, GC–MS/MS, HRMS (Orbitrap/QTOF) with minimal cleanup	Better integration with modern high-resolution systems	[3,72]

## Data Availability

No new data generated or analyzed.

## Abbreviations

The following abbreviations are used in this manuscript:

AGREE	Analytical GREEnness
CS2	Carbon disulfide
DES	Deep eutectic solvents
DI-SPME	Direct immersion solid-phase microextraction
DLLME	Dispersive liquid–liquid microextraction
DMI	Demethylation inhibitor
d-SPE	Dispersive solid-phase extraction
ECD	Electron capture detector
EFSA	European Food Safety Authority
EMR-Lipid	Enhanced matrix removal–lipid
ETU	Ethylene thiourea
GAC	Green Analytical Chemistry
GC	Gas chromatography
GC-MS	Gas chromatography–mass spectrometry
GC-MS/MS	Gas chromatography–tandem mass spectrometry
GCB	Graphitized carbon black
HF-LPME	Hollow-fiber liquid-phase microextraction
HRMS	High-resolution mass spectrometry
HS-SPME	Headspace solid-phase microextraction
HPLC	High-performance liquid chromatography
IDMS	Isotope dilution mass spectrometry
IL	Ionic liquid
LC	Liquid chromatography
LC-HRMS	Liquid chromatography–high-resolution mass spectrometry
LC-MS/MS	Liquid chromatography–tandem mass spectrometry
LLE	Liquid–liquid extraction
LOQ	Limit of quantification
LPME	Liquid-phase microextraction
MeCN	Acetonitrile
MOF	Metal–organic framework
MRL	Maximum residue limit
MRM	Multiple reaction monitoring
MS	Mass spectrometry
MS/MS	Tandem mass spectrometry
MSPE	Magnetic solid-phase extraction
MSTFA	N-methyl-N-trimethylsilyl-trifluoroacetamide
NTS	Non-target screening
PSA	Primary–secondary amine
PT-PSE	Pipette-tip solid-phase extraction
PTU	Propylene thiourea
Q-TOF	Quadrupole time-of-flight
QqQ	Triple quadrupole
QoI	Quinone outside inhibitor
QuEChERS	Quick, Easy, Cheap, Effective, Rugged, and Safe
SDHI	Succinate dehydrogenase inhibitor
SDME	Single-drop microextraction
SEA	Southeast Asia
SBSE	Stir bar sorptive extraction
sMRM	Scheduled multiple reaction monitoring
SPE	Solid-phase extraction
SPME	Solid-phase microextraction
TOF	Time-of-flight
UHPLC	Ultra-high-performance liquid chromatography
UPHLC-MS/MS	Ultra-high-performance liquid chromatography–tandem mass spectrometry
UPLC	Ultra-performance liquid chromatography
UPLC-Orbitrap MS	Ultra-performance liquid chromatography–Orbitrap mass spectrometry
UPLC-MS/MS	Ultra-performance liquid chromatography–tandem mass spectrometry
UAME	Ultrasound-assisted microextraction
